# Genomic Screening for Pathogenic Transthyretin Variants Finds Evidence of Underdiagnosed Amyloid Cardiomyopathy From Health Records

**DOI:** 10.1016/j.jaccao.2021.07.002

**Published:** 2021-10-19

**Authors:** Brendan J. Carry, Katelyn Young, Samuel Fielden, Melissa A. Kelly, Amy C. Sturm, J. David Avila, Christa L. Martin, H. Lester Kirchner, Brandon K. Fornwalt, Christopher M. Haggerty, Regeneron Genetics Center, Tarrytown, New York, USA

**Affiliations:** a Heart Institute, Geisinger Medical Center, Danville, Pennsylvania, USA; b Department of Internal Medicine, Geisinger Medical Center, Danville, Pennsylvania, USA; c Department of Translational Data Science and Informatics, Geisinger Medical Center, Danville, Pennsylvania, USA; d Genomic Medicine Institute, Geisinger Medical Center, Danville, Pennsylvania, USA; e Department of Neurology, Geisinger Medical Center, Danville, Pennsylvania, USA; f Autism & Developmental Medicine Institute, Geisinger Medical Center, Danville, Pennsylvania, USA; g Department of Population Health Sciences, Geisinger Medical Center, Danville, Pennsylvania, USA; h Department of Radiology, Geisinger Medical Center, Danville, Pennsylvania, USA

**Keywords:** genomics, amyloidosis, electronic health records, cardiomyopathy, ATTR, transthyretin amyloidosis, CI, confidence interval, EHR, electronic health record, hATTR, hereditary transthyretin amyloidosis, HCC, hierarchical condition categories, LV, left ventricle/ventricular, OR, odds ratio, P, pathogenic, LP, likely pathogenic, TTR, transthyretin

## Abstract

**Background:**

New treatments for transthyretin amyloidosis improve survival, but diagnosis remains challenging. Pathogenic or likely pathogenic (P/LP) variants in the transthyretin (*TTR*) gene are one cause of transthyretin amyloidosis, and genomic screening has been proposed to identify at-risk individuals. However, data on disease features and penetrance are lacking to inform the utility of such population-based genomic screening for *TTR.*

**Objectives:**

This study characterized the prevalence of P/LP variants in *TTR* identified through exome sequencing and the burden of associated disease from electronic health records for individuals with these variants from a large (N = 134,753), primarily European-ancestry cohort.

**Methods:**

We compared frequencies of common disease features and cardiac imaging findings between individuals with and without P/LP *TTR* variants.

**Results:**

We identified 157 of 134,753 (0.12%) individuals with P/LP *TTR* variants (43% male, median age 52 [Q1-Q3: 37–61] years). Seven P/LP variants accounted for all observations, the majority being V122I (p.V142I; 113, 0.08%). Approximately 60% (n = 91) of individuals with P/LP *TTR* variants (all V122I) had African ancestry. Diagnoses of amyloidosis were limited (2 of 157 patients), although related heart disease diagnoses, including cardiomyopathy and heart failure, were significantly increased in individuals with P/LP *TTR* variants who were aged >60 years. Fourteen percent (7 of 49) of individuals aged ≥60 or older with a P/LP *TTR* variant had heart disease and ventricular septal thickness >1.2 cm, only one of whom was diagnosed with amyloidosis.

**Conclusions:**

Individuals with P/LP *TTR* variants identified by genomic screening have increased odds of heart disease after age 60 years, although amyloidosis is likely underdiagnosed without knowledge of the genetic variant.

Transthyretin amyloidosis (ATTR) encompasses a group of systemic diseases characterized by the extracellular accumulation of insoluble transthyretin (also known as prealbumin) fibrils (1). Although many cases are attributed to age-related misaggregation of genetically normal (“wild-type”) transthyretin, pathogenic or likely pathogenic (P/LP) variants in the transthyretin gene (*TTR*) are also known to cause protein misfolding, leading to hereditary disease (hATTR) (2). Most commonly, hATTR clinically manifests as polyneuropathy and/or cardiomyopathy, with an estimated prevalence of 1:100,000 in the United States (3). However, this prevalence is likely underestimated due to the challenges of diagnosing patients with hATTR-associated cardiomyopathy and polyneuropathy (4).

The advent of more widespread genetic testing facilitated by next-generation DNA sequencing technologies presents new opportunities to potentially redefine our understanding and approach to hATTR diagnosis and treatment. Identifying individuals at risk through systematic screening for genomic variants in *TTR*, rather than symptom-based clinical ascertainment, could both clarify the true scope of the disease at a population scale and enable earlier intervention to potentially mitigate disease progression and thereby improve outcomes (5). Because hATTR cardiomyopathy is generally only clinically recognized once profound ventricular remodeling and dysfunction are present these opportunities for improving outcomes may be substantial, particularly in light of newly approved treatments (1,6). In fact, the Clinical Genome Resource Actionability Working Group has recently upgraded its assertion for *TTR* and cardiac amyloidosis to “strong actionability” (7); however, they do note that there is limited evidence informing disease likelihood and intervention effectiveness in this context. Moreover, *TTR* is not yet included in most secondary finding or population-based screening analyses, such as the American College of Medical Genetics and Genomics secondary findings gene list, supporting the need for additional data (8).

To establish the potential efficacy of this genome-first approach, it is first important to understand the population prevalence of P/LP variants in *TTR* and how they present clinically, independent of a symptoms-based ascertainment. For example, Damrauer et al (9) recently showed significantly increased odds of heart failure in genetically ascertained individuals of African ancestry older than the age of 50 years with a specific *TTR* variant—V122I, also known as p.V142I (9). This variant is common (up to 4%) in individuals of African ancestry, with at least one study finding an incidence of heart failure of 29% over 21.5 years of follow-up (10, 11, 12). This finding thus provides strong support for the premise of genomic screening for *TTR* variants. However, as of April 2021, there are 67 other known P/LP variants in *TTR* listed in the National Center for Biotechnology Information ClinVar database*,* and for the remaining majority, little is known about the phenotype associations outside of symptoms-based clinical ascertainment (13). Therefore, the objective of the present study was to evaluate the phenotypic associations of all observed P/LP variants in *TTR* within a large, population-based health care cohort using research exome sequencing paired with electronic health record (EHR) data. We hypothesized that such variants would be associated with cardiac, neuropathic, and ophthalmologic phenotypes, and specific abnormalities from diagnostic testing through genomic screening-based identification.

## Methods and Materials

Institutional review board approval was obtained for this study; all individuals previously provided informed consent to participate in the Geisinger MyCode Community Health Initiative.

### Cohort

The MyCode Community Health Initiative at Geisinger is a precision health project (2007–present) in which participants, with opt-in informed consent, provide biospecimens for broad research use, including permission to link associated data to the EHR. Exome sequencing of blood or saliva samples collected from MyCode participants was performed through the DiscovEHR collaboration between Geisinger and the Regeneron Genetics Center (Tarrytown, New York). This study included the first 145,454 participants who underwent sequencing, comprising a predominantly adult population of European ancestry.

### Genetic sequencing, variant calling and annotation, and genotype assignment

Genomic DNA was isolated from patients’ blood or saliva. Exome sequencing was performed in collaboration with Regeneron Genetics Center as previously described (14). Probes from Nimblegen (VCRome) or a version of the xGEN probe from Integrated DNA Technologies were used for target sequence capture. Sequencing was performed by paired end 75bp reads on either an Illumina HiSeq2500 or NovaSeq. Coverage depth was sufficient to provide more than 20% coverage over 85% of the targeted bases in 96% of the VCRome samples and 90% coverage for 99% of Integrated DNA Technologies samples. Following sequencing, samples showing disagreement between genetically determined and reported sex, low-quality sequence data, samples with contamination, genetically identified sample duplicates, and samples with discordance between exome and genotype results were removed, leaving sequence data for 144,204 participants available for analysis.

Alignments and variant calling were based on GRCh38 human genome reference sequence. Variant calls were produced using the WeCall variant caller (Genomics PLC). A project-level variant-call file was compiled using the GLnexus joint genotyping tool (version 1.1.3-4). From that file, we selected all nonsynonymous single nucleotide variants in *TTR* based on variant effect annotated by ANNOVAR, with a minor allele frequency <0.001 within MyCode and at least a 1∗ pathogenic or likely pathogenic classification in ClinVar (as of 20210123 database) (15). Sample-level variant calls with site read depth <7, and alternate allele balance <15% (or fewer than 5 alternate reads) were removed.

### Phenotype evaluation

We collected available demographic (date of birth, sex, age at last encounter, and vital status), diagnostic (International Classification of Diseases [ICD]-9th or ICD-10th Revision codes from clinical encounters and patient problem lists), and clinical findings from the most recent echocardiogram, as available as of February 2020. Analysis was restricted to individuals with at least 4 weeks of longitudinal follow-up between their first and last encounters in the EHR (N = 134,753).

Cardiac amyloidosis was defined using diagnoses of “amyloid heart disease (hierarchical condition categories [HCC]),” “cardiac amyloidosis (HCC),” and “restrictive cardiomyopathy secondary to amyloidosis (HCC).” General (noncardiac-specific) amyloidosis diagnoses (inclusive of amyloid neuropathy) were defined based on E85 ICD-10 codes (Supplemental Table 1). Other prespecified cardiac (cardiomyopathy [specifically nonischemic etiology and excluding dilated presentation], heart failure, atrial fibrillation, aortic valve stenosis, atrioventricular block, bundle branch block, and sick sinus syndrome), peripheral neuropathy (carpal tunnel syndrome, spinal stenosis, limb mononeuropathy, unspecified mononeuropathy, or polyneuropathy), autonomic neuropathy (autonomic dysfunction, incontinence, or impotence), ophthalmological (cataracts or glaucoma), or miscellaneous (hepatomegaly) phenotypes of interest were defined using diagnosis codes or custom definitions, as detailed in Supplemental Table 1. Diagnoses were considered valid if the specified codes were used on 2 or more clinical encounters or listed as active on the patient’s problem list. Other diagnostic findings of interest included left ventricular (LV) wall thickness, LV cavity size, or ejection fraction from the most recent echocardiography exam.

### Statistics

Categorical data are reported as number (percentage); continuous data are presented as median [25th–75th percentiles] ([Q1–Q3]) or mean ± SD, as indicated. Statistical analyses were performed using R, version 4.3. To compare the prevalence of diagnoses of interest between individuals with P/LP *TTR* variants and the rest of the sequenced cohort, we used Firth’s bias-reduced logistic regression for binary outcomes with small samples (using the “logistf” package in R) (16). Robust linear regression was similarly used to compare quantitative echocardiography measures. To account for population relatedness, we used a bootstrap procedure (1,000 iterations, sampling at the family level based on identity-by-descent estimation) to estimate the variance in the regression coefficient (17). All models were adjusted for age (and age^2^) at most recent encounter (or at the time of the echocardiogram), sex, and the first 4 principal components of ancestry. Model results are presented as odds ratios (ORs) for logistic regression or regression coefficients (β) for linear regression and 95% confidence intervals (CIs) for both. For relatedness-adjusted variant population prevalence estimates, individuals with closer than third-degree relatedness were excluded. All *P* values were adjusted for multiple comparisons using the Benjamini-Hochberg false-discovery rate. A value of *P* < 0.05 was considered statistically significant.

## Results

### Prevalence and demographics of individuals with P/LP *TTR* variants

We observed a total of 157 individuals (0.12%) carrying a P/LP variant in *TTR*, the majority (113 of 157 patients, 72%) of whom had the V122I variant (Central Illustration). Table 1 details the observed variants, all of which had a 2∗ ClinVar classification (manual confirmation as of April 2021). One individual was homozygous for V122I (31-year-old female with an unremarkable EHR phenotype), all other individuals were heterozygous for their respective variant. Approximately 60% of the individuals with P/LP *TTR* variants had African ancestry (91 of 157 patients), all of whom had the V122I variant, reaffirming the variant’s high frequency in that population (91 of 3,459, 2.6%). Table 2 summarizes the demographic characteristics of individuals with P/LP *TTR* variants and the rest of the cohort, including race-specific details for the African-ancestry subset.

**Central Illustration:**
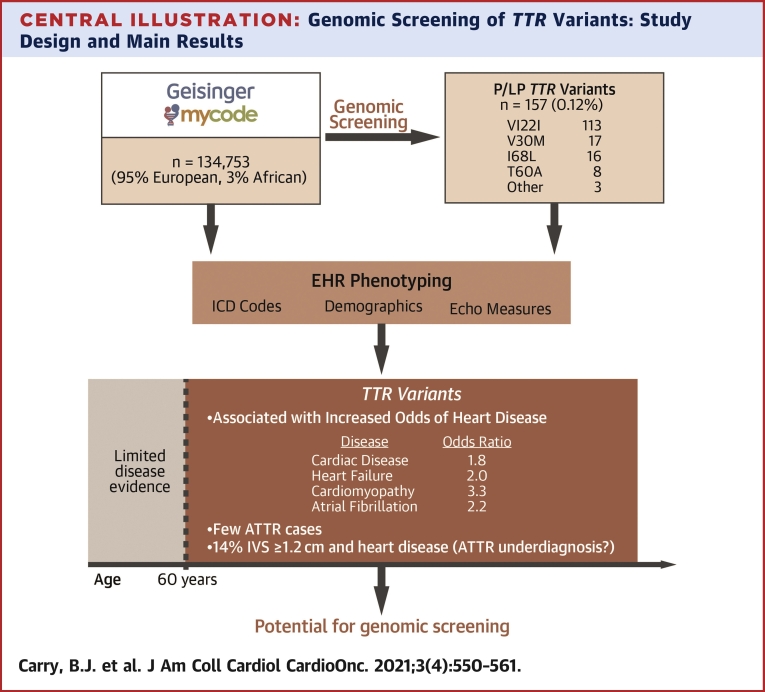
Genomic Screening of *TTR* Variants: Study Design and Main Results Of 134,753 individuals, 157 were identified with pathogenic/likely pathogenic (P/LP) variants in the transthyretin (*TTR*) gene from MyCode, which is a large clinical population of predominantly European ancestry. Via linked electronic health records (EHRs), we found significantly increased odds of heart disease in individuals aged ≥60 years, despite few diagnoses of transthyretin amyloidosis (ATTR). Genomic screening of *TTR* may therefore improve disease recognition in a clinical setting. ICD = International Classification of Diseases.

**Table 1:** Observed P/LP Variants in *TTR* in MyCode (N = 157)

Variant	HGVSc	HGVSp	n	Population Prevalence^a^	Unrelated Population Prevalence^a^^,^^b^
V30M	NM_000371:c.148G>A	p.Val50Met	17	1:8,013	1:6,525
F33L	NM_000371:c.157T>C	p.Phe53Leu	1	1:128,213	1:78,301
L58H	NM_000371:c.233T>A	p.Leu78His	1	1:128,213	1:78,301
T60A	NM_000371:c.238A>G	p.Thr80Ala	8	1:16,027	1:9,788
I68L	NM_000371:c.262A>T	p.Ile88Leu	16	1:8,013	1:9,788
I107V	NM_000371:c.379A>G	p.Ile127Val	1	1:128,213	1:78,301
V122I	NM_000371:c.424G>A	p.Val142Ile	113	1:9,158 (EUR) 1:38 (AFR)	1:7,118 (EUR) 1:37 (AFR)

AFR = African ancestry; EUR = European ancestry; LP = likely pathogenic; P = pathogenic; *TTR* = transthyretin.

a Prevalence within MyCode for European-ancestry individuals (EUR) only, unless otherwise specified.

b Excluding individuals with closer than third-degree relatedness.

**Table 2:** Characteristics of Individuals With P/LP *TTR* Variant and the Remaining MyCode Cohort

	Individuals With *TTR*_P/LP_ (All) (N = 157, 0.12%)	MyCode Participants, Without *TTR*_P/LP_ (All) (N = 134,596)	Individuals With *TTR*_P/LP_ (African Ancestry) (N = 91, 2.6%)	MyCode Participants, Without *TTR*_P/LP_ (African Ancestry) (N = 3,368)
Male	67 (43)	52,347 (39)	41 (45)	1,168 (35)
EUR	57 (36)	128,156 (95)	0	0
Time in her, y	11 [5-15]	15 [9-18]	9 [5-15]	8 [4-13]
Age at last encounter, y	52 [37–61]	58 [43–70]	49 [33–59]	42 [30-56]
Body mass index, kg/m^2^	32 [27-37]	30 [26-35]	32 [28-38]	32 [27-37]
Vital status, % alive	94	91	98	97

Values are n (%) or median [Q1-Q3].

HER = electronic health record; *TTR*_P/LP_ = pathogenic/likely pathogenic variant in *TTR*; other abbreviations as in Table 1.

### Disease associations of the full cohort

Data for phenotype observations and statistical comparisons with controls (ie, sequenced MyCode participants without a P/LP *TTR* variant) are detailed in Table 3. One individual (1 of 157 patients; 0.64%) with a P/LP *TTR* variant (T60A) had an existing diagnosis of cardiac amyloidosis compared with 31 individuals without a P/LP *TTR* variant (31 of 134 patients, 596; 0.02%). Two individuals (2 of 157; 1.3%) with a P/LP *TTR* variant (T60A [same patient as above] and V122I) had a diagnosis of noncardiac-specific (general) amyloidosis compared to 136 controls without a P/LP *TTR* variant (136 of 134,596; 0.1%). By cross-referencing medication orders and reconciliations, we confirmed that no additional individuals with P/LP *TTR* variants were prescribed ATTR therapy—specifically tafamidis, patisiran, inotersen, or diflunisal—to raise suspicion of diagnosed amyloidosis. In the full cohort—without any age restriction—no significant associations were observed between P/LP *TTR* variants and cardiac, peripheral or autonomic neuropathy, ophthalmological, or other phenotypes after adjusting for multiple testing.

**Table 3:** Phenotype Associations for All P/LP Variants in *TTR*

	*TTR*_P/LP_ (n = 157)	Control (Without *TTR*_P/LP_) (n = 134,596)	OR (95% CI)	*P* Value
Amyloidosis				
Cardiac amyloidosis	1 (0.6)	31 (0.02)	NA^a^	
Amyloidosis, general	2 (1.3)	136 (0.1)	NA	
Cardiac	24 (15.0)	24,599 (18.0)	1.4 (0.9-1.9)	0.23
Heart failure	14 (9.0)	11,943 (9.0)	1.5 (1.0-2.1)	0.19
Cardiomyopathy	7 (4.0)	3,407 (3.0)	1.7 (0.9-2.9)	0.21
Atrial fibrillation	13 (8.0)	13,813 (10.0)	1.9 (1.1-3.0)	0.089
Aortic valve stenosis	5 (3.0)	5,210 (4.0)	2.2 (1.0-3.8)	0.089
Atrioventricular block	1 (0.6)	1,979 (1.5)	0.9 (0.4-1.9)	0.86
Bundle branch block	4 (3.0)	2,795 (2.0)	2.1 (0.9-3.8)	0.21
Sick sinus syndrome	2 (1.0)	1,396 (1.0)	3.9 (1.0-8.3)	0.089
Peripheral neuropathy	20 (13.0)	22,460 (17.0)	0.8 (0.6-1.2)	0.51
Carpal tunnel	12 (8.0)	11,287 (8.0)	1.1 (0.6-1.6)	0.86
Spinal stenosis	9 (6.0)	9,818 (7.0)	1.1 (0.6-1.8)	0.82
Limb mononeuropathy/unspecified mononeuropathy or polyneuropathy	5 (3.0)	5,080 (4.0)	0.8 (0.3-1.3)	0.68
Autonomic neuropathy	25 (16.0)	22,125 (16.0)	1.2 (0.8-1.7)	0.51
Autonomic dysfunction^b^	3 (2.0)	3,208 (2.0)	1.3 (0.3-2.3)	0.80
Incontinence	8 (5.0)	11,653 (9.0)	0.6 (0.1-1.1)	0.49
Impotence	15 (10.0)	8,619 (6.0)	1.4 (0.8-2.2)	0.28
Ophthalmological	17 (11.0)	21,796 (16.0)	1.0 (0.6-1.4)	0.86
Cataract	17 (11.0)	21,115 (16.0)	1.0 (0.6-1.5)	0.86
Glaucoma	1 (0.6)	2,842 (2.0)	0.5 (0.2-1.1)	0.47
Miscellaneous				
Hepatomegaly	2 (1.0)	778 (0.6)	3.3 (1.0-6.1)	0.089
Multisystem				
Cardiac + other system^c^	13 (8.0)	14,545 (11.0)	1.3 (0.8-2.0)	0.47

Values are n (%) unless otherwise indicated. OR and *P* values based on logistic regression model adjusted for age, age^2^, sex, and principal components 1–4 of ancestry; 95% CI and *P* values based on bootstrapped sampling procedure; Reported *P* values are adjusted to control false discovery rate.

CI = confidence interval; NA = not available; OR = odds ratio; other abbreviations as in Tables 1 and 4.

a OR and *P* values not reported due to limited observations.

b Includes gastroparesis, orthostatic hypotension, and eccrine sweat disorder.

c Other system denotes any peripheral neuropathy, autonomic neuropathy, ophthalmology, or miscellany.

### Quantitative findings of the full cohort

Prior echocardiograms to assess LV function were available for 61,595 individuals (46%), although this frequency was lower for the *TTR* group (34%, *P* = 0.003). Diastolic septal thickness was slightly increased in the individuals with P/LP *TTR* variants (1.15 ± 0.24 cm vs 1.08 ± 0.24 cm), although the difference was not significant after adjusting for age, sex, and ancestry (β = 0.06, *P* = 0.076). Similarly, there were no significant differences in other left ventricular structural metrics (chamber volume or posterior wall thickness) or function (ejection fraction) based on *TTR* variant status (Table 4).

**Table 4:** Groupwise Comparisons of Select Echocardiography-derived Measures Between Individuals With P/LP *TTR* Variants and Controls

N_TTR_/N_Control_	*TTR*_P/LP_	Control (Without *TTR*_P/LP_)	β (95% CI)	*P* Value
IVSd, cm (51/60,326)	1.2 ± 0.2	1.1 ± 0.2	0.06 (0.01 to 0.10)	0.076
LVPWd, cm (50/60,225)	1.1 ± 0.2	1.0 ± 0.2	0.04 (-0.01 to 0.09)	0.36
IVS/LVPW (50/59,639)	1.1 ± 0.2	1.1 ± 0.3	0.02 (-0.01 to 0.04)	0.36
LVIDd, cm (49/60,510)	4.7 ± 0.6	4.7 ± 0.7	0.01 (-0.10 to 0.14)	0.89
EF, % (53/61,542)	56.3 ± 9.1	56.9 ± 9.1	-0.36 (-1.57 to 0.60)	0.64

Values are mean ± 1 SD unless otherwise indicated. β coefficients and *P* values based on linear regression model adjusted for age, age^2^, sex, and principal components 1–4 of ancestry. 95% CI and *P* values based on bootstrapped sampling procedure. Reported *P* values are adjusted to control false discovery rate. β coefficients represent the difference between *TTR* group and controls in echocardiography-derived measure of interest.

EF = ejection fraction; IVS(d) = interventricular septum thickness at end-diastole; LVIDd = left ventricular internal diameter at end-diastole; LVPW(d) = left ventricular posterior wall thickness at end-diastole; other abbreviations as in Table 1, Table 2 and Table 3.

### Age-stratified subanalysis

Hypothesizing that the lack of significant disease associations with P/LP *TTR* variants, particularly with cardiac phenotypes, was in part mediated by age-dependent penetrance of these conditions, we subset the cohort to separately evaluate individuals ≥60 years of age. This cutoff was selected for consistency with prior analyses and based on frequently reported age at symptom onset (11,18,19). This analysis thus included 64,316 individuals, including 49 with a P/LP *TTR* variant (Supplemental Table 2); results are shown in Table 5.

**Table 5:** Phenotype Associations for All P/LP Variants in *TTR* in Subset of Individuals 60 Years of Age or Older

	*TTR*_P/LP_ (n = 49)	Control (Without *TTR*_P/LP_) (n = 64,267)	OR (95% CI)	*P* Value
Amyloidosis				
Cardiac amyloidosis	1 (2.0)	30 (0.05)	NA^a^	
Amyloidosis, general	1 (2.0)	113 (0.2)	NA	
Cardiac	17 (35.0)	20,756 (32.0)	1.8 (1.1-2.8)	0.039
Heart failure	11 (22.0)	10,385 (16.0)	2.0 (1.2-3.1)	0.016
Cardiomyopathy	6 (12.0)	2,511 (4.0)	3.3 (1.2-6.0)	0.012
Atrial fibrillation	10 (20.0)	12,271 (19.0)	2.2 (1.1-4.0)	0.036
Aortic valve stenosis	5 (10.0)	4,743 (7.0)	2.9 (1.2-5.2)	0.016
Atrioventricular block	1 (2.0)	1,760 (2.7)	1.4 (0.6-3.4)	0.81
Bundle branch block	4 (8.0)	2,291 (4.0)	3.6 (0.8-7.0)	0.023
Sick sinus syndrome	2 (4.0)	1,318 (2.0)	4.5 (1.2-10.0)	0.022
Peripheral neuropathy	13 (27.0)	14,425 (22.0)	1.2 (0.7-2.0)	0.68
Carpal tunnel	7 (14.0)	6,346 (10.0)	1.5 (0.7-2.5)	0.45
Spinal stenosis	8 (16.0)	7,698 (12.0)	1.6 (0.8-2.8)	0.25
Limb mononeuropathy / unspecified mononeuropathy or polyneuropathy	3 (6.0)	3,162 (5.0)	1.3 (0.3-2.6)	0.81
Autonomic neuropathy	11 (22.0)	14,946 (23.0)	1.0 (0.5-1.6)	0.99
Autonomic dysfunction^b^	1 (2.0)	2,093 (3.0)	1.0 (0.4-2.2)	0.99
Incontinence	5 (10.0)	7,601 (12.0)	1.0 (0.2-1.9)	0.99
Impotence	5 (10.0)	6,318 (10.0)	0.9 (0.3-1.8)	0.99
Ophthalmological	14 (29.0)	19,863 (31.0)	1.1 (0.6-1.9)	0.99
Cataract	14 (29.0)	19,336 (30.0)	1.2 (0.6-2.0)	0.81
Glaucoma	0	2,563 (4.0)	—	—
Miscellaneous				
Hepatomegaly	0	310 (0.5)	—	—
Multisystem				
Cardiac + other system^c^	11 (22.0)	13,117 (20.0)	1.7 (0.9-2.8)	0.18

Values are n (%) unless otherwise indicated. OR and *P* values based on logistic regression model adjusted for age, age^2^, sex, and principal components 1–4 of ancestry. 95% CI and *P* values based on bootstrapped sampling procedure. Reported *P* values are adjusted to control false-discovery rate.

Abbreviations as in Tables 1 and 3.

a OR and *P* values not reported due to limited observations.

b Includes gastroparesis, orthostatic hypotension, and eccrine sweat disorder.

c Other system denotes any peripheral neuropathy, autonomic neuropathy, ophthalmology, or miscellany.

Consistent with our hypothesis, we found that 17 of 49 (35%) patients of this older subset had at least 1 cardiac phenotype, representing a significant increase from control (OR: 1.8; 95% CI: 1.1–2.8; *P* = 0.039). These findings included increased risk for diagnoses of cardiomyopathy (OR: 3.3; 95% CI: 1.2-6.0; *P* = 0.012), heart failure (OR: 2.0; 95% CI: 1.2-3.1; *P* = 0.016), and atrial fibrillation (OR: 2.2; 95% CI: 1.1-4.0; *P* = 0.036) (Table 5). Moreover, these diagnostic findings coincided with nonsignificant association with increased septal (1.20 ± 0.23 cm vs. 1.13 ± 0.24 cm; *P* = 0.075) and posterior wall thicknesses (1.18 ± 0.26 cm vs. 1.07 ± 0.21 cm; *P* = 0.075) (Table 6). Moreover, 7 of 49 (14%) of these individuals with *TTR* variants aged 60 years or older had both a diagnosis of heart disease and a septal thickness ≥1.2 cm by echocardiography. This combination is strongly suggestive of penetrant cardiac amyloidosis, although only 1 of these individuals had a clinical diagnosis of amyloidosis.

**Table 6:** Group-wise Comparisons of Select Echocardiography-derived Measures Between the Subset of Individuals With P/LP *TTR* Variants and Controls Aged 60 Years or Older at the Time of the Study

(N_TTR_/N_Control_)	*TTR*_P/LP_	Control (Without *TTR*_P/LP_)	β (95% CI)	*P* Value
IVSd, cm (20/34,747)	1.20 ± 0.23	1.13 ± 0.24	0.09 (0.01 to 0.17)	0.075
LVPWd, cm (19/34,673)	1.18 ± 0.26	1.07 ± 0.21	0.12 (0.02 to 0.21)	0.075
IVS/LVPW (19/34,472)	1.03 ± 0.07	1.07 ± 0.07	-0.02 (-0.04 to 0.01)	0.31
LVIDd, cm (18/34,843)	4.63 ± 0.73	4.62 ± 0.75	0.05 (-0.20 to 0.37)	0.72
EF, % (22/35,878)	53.1 ± 12.3	56.1 ± 10.4	-2.00 (-4.65 to 0.32)	0.17

Values are mean ± 1 SD unless otherwise indicated. β coefficients and *P* values based on linear regression model adjusted for age, age^2^, sex, and principal components 1–4 of ancestry. 95% CI and *P* values based on bootstrapped sampling procedure. Reported *P* values are adjusted to control false discovery rate. β coefficients represent the difference between *TTR* group and controls in echocardiography-derived measure of interest.

Abbreviations as in Table 1, Table 2, Table 3 and Table 4.

In contrast, the burden of noncardiac phenotypes was no different from the rest of the population, and the odds of multisystem diagnoses (cardiac and neuropathy/ophthalmological/miscellaneous) were not different based on *TTR* variant status.

### Subanalysis of V122I

In the subset of individuals with V122I (median [Q1-Q3]: 49 [33–59] years), we observed significantly increased odds of atrial fibrillation (OR: 2.3; 95% CI: 1.2-3.9; *P* = 0.032), sick sinus syndrome (OR: 10.9; 95% CI: 3.1-27.1; *P* < 0.001), and hepatomegaly (OR: 5.1; 95% CI: 1.4-10.5; *P* = 0.017) (Table 7). Only 1 individual (0.89%) had an existing diagnosis of amyloidosis, again suggestive of underdiagnosis. Odds of observed neuropathy were no different for individuals with V122I.

**Table 7:** Phenotype Associations for Individuals With the V122I Variant Compared With the Rest of the MyCode Cohort

	*TTR*_P/LP_ (n = 113)	Control (Without V122I) (n = 134,640)	OR (95%CI)	*P* Value
Amyloidosis				
Cardiac amyloidosis	0	31 (0.02)	—	—
Amyloidosis, general	1 (0.9)	137 (0.1)	NA^a^	
Cardiac	16 (14.0)	24,607 (18.0)	1.5 (0.9-2.3)	0.32
Heart failure	7 (6.0)	11,950 (9.0)	1.1 (0.5-1.8)	0.86
Cardiomyopathy	4 (4.0)	3,410 (3.0)	1.3 (0.6-2.4)	0.80
Atrial fibrillation	8 (7.0)	13,818 (10.0)	2.3 (1.2-3.9)	0.032
Aortic valve stenosis	2 (2.0)	5,213 (4.0)	2.2 (0.7-4.2)	0.32
Atrioventricular block	1 (1.0)	1,979 (1.0)	1.4 (0.6-3.0)	0.80
Bundle branch block	3 (3.0)	2,796 (2.0)	2.6 (0.6-5.0)	0.20
Sick sinus syndrome	2 (2.0)	1,396 (1.0)	10.9 (3-27)	<0.001
Peripheral neuropathy	15 (13.0)	22,465 (17.0)	0.9 (0.6-1.4)	0.81
Carpal tunnel	9 (8.0)	11,290 (8.0)	1.2 (0.6-1.8)	0.80
Spinal stenosis	6 (5.0)	9,821 (7.0)	1.2 (0.5-2.0)	0.80
Limb mononeuropathy/unspecified mononeuropathy or polyneuropathy	4 (4.0)	5,081 (4.0)	0.8 (0.3-1.4)	0.80
Autonomic neuropathy	17 (15.0)	22,133 (16.0)	1.2 (0.8-1.8)	0.60
Autonomic dysfunction^b^	3 (3.0)	3,208 (2.0)	2.1 (0.4-3.9)	0.32
Incontinence	3 (3.0)	11,658 (9.0)	0.6 (0.1-1.1)	0.58
Impotence	12 (11.0)	8,622 (6.0)	1.5 (0.8-2.5)	0.32
Ophthalmological	7 (6.0)	21,806 (16.0)	0.6 (0.3-1.0)	0.32
Cataract	7 (6.0)	21,125 (16.0)	0.6 (0.3-1.1)	0.39
Glaucoma	0	2,843 (2.0)	—	—
Miscellaneous				
Hepatomegaly	2 (2.0)	778 (0.6)	5.1 (1.4-10.5)	0.017
Multisystem				
Cardiac + other system^c^	7 (6.0)	14,551 (11.0)	1.2 (0.5-2.1)	0.81

Values are n (%). OR and *P* values based on logistic regression model adjusted for age, age^2^, sex, and principal components 1–4 of ancestry. 95% CI and *P* values based on bootstrapped sampling procedure. Reported *P* values are adjusted to control false-discovery rate.

Abbreviations as in Tables 1 and 3.

a OR and *P* values not reported due to limited observations.

b Includes gastroparesis, orthostatic hypotension, and eccrine sweat disorder.

c Other system denotes any peripheral neuropathy, autonomic neuropathy, ophthalmology, or miscellany.

## Discussion

Most studies of the *TTR* gene and its associated phenotypes have focused on patients ascertained through symptoms-based clinical presentation and/or carrying a specific *TTR* variant (9,10,19, 20, 21). This study is uniquely centered on our population-based genome-first ascertainment from a large, predominantly rural, U.S. health care system, the size of the available sequenced cohort (n = 144,204), and the inclusion of all observed P/LP *TTR* variants. In this setting—a cohort with predominantly European ancestry—the overall prevalence of P/LP *TTR* variants was 0.12%. Extrapolating this observation to match estimates of US racial demographics yields a prevalence of 0.45%—driven by the high frequency of V122I (2.6% in our data) among African-ancestry individuals—which is considerably more common than current disease estimates (0.001% in the United States) (3). Consistent with this variant/disease prevalence mismatch, clinical diagnosis of amyloidosis (both cardiac and general/noncardiac) was uncommon in our population. The data reveal that the presence of these variants in the population is associated with increased risk of heart disease, suggesting that ATTR cardiomyopathy is underdiagnosed. For individuals 60 years of age or older, we observed significantly increased odds of relevant cardiac phenotypes with P/LP *TTR* variants, particularly cardiomyopathy, heart failure, and atrial fibrillation. In at least 14% of individuals with echocardiography data, these diagnoses presented with increased ventricular wall thickness, which is strongly suggestive of penetrant ATTR. In only 1 of these patients was the diagnosis of ATTR applied, providing the opportunity for optimal therapy. Collectively, these results provide support for the use of genomic screening for P/LP *TTR* variants in population settings, particularly for communities with a large proportion of African-ancestry individuals. The findings also show the importance of considering age in the potential actionability of these variants. Heart disease risk in this cohort comprising primarily individuals with the V122I variant was only observed to be significant in individuals 60 years of age or older. Screening before expected age of onset would be appropriate with the goal of preventing disease development or progression, but the optimal timing of potential intervention must be identified on a variant-specific basis.

Despite the relatively small representation of African Americans in MyCode (2.5%), V122I was the most commonly observed P/LP *TTR* variant in our cohort, exhibiting a prevalence of 2.6% to 2.7% within African-ancestry individuals, similar to published literature (21,22). By comparison, the V122I prevalence was 0.014% in individuals of European ancestry, which is consistent with prior work (0 to 0.028%), and the ancestry-specific allele frequency (0.00003) reported in gnomAD v.2.1.1 (10). Our three next most common variants were observed in 1 of 6,581 (0.015%; V30M), 1 of 9,872 (0.010%; I68L), and 1 of 9,872 (0.010%; T60A). Comparatively, a recent analysis of gnomAD showed the following prevalence in a similar ethnically European population: V30M (1 of 2,792; 0.036%), I68L (1 of 12,668; 0.008%), and T60A (1 of 55,842; 0.002%) (23). Notably, our prevalence of T60A carriers is more than 5 times higher than gnomAD, which could reflect regional differences in ancestry subpopulations, such as a high concentration of Irish immigrants to the Appalachian region (24,25).

### Age-dependent penetrance of *TTR* variants

The age dependence of *TTR*-associated disease is well recognized. In fact, varying age-of-onset characteristics have even been documented between the various “common” *TTR* variants, generally reflecting onset between the 5th and 7th decades of life (1). For example, Sattianayagam *et al* (19) reported a median age of symptom development—a mix of neuropathy and cardiomyopathy—as 63 years in a small cohort of patients with the T60A variant. Variants with multiple phenotype associations (eg, V30M) have been described as having bimodal presentation, with early onset of neuropathy (median of 42 years), and later onset of mixed neuropathy and cardiomyopathy (median of 53 years) (20). Buxbaum et al (21) documented the characteristics of Black Americans with V122I in the Arteriosclerosis Risk In Communities (ARIC) and Cardiovascular Health (CHS) studies, finding increased risk of heart failure and mortality after 65 years, but no discernable impact prior to that age (21). Finally, retrospective analysis of Black American patients older than age 60 years with New York Heart Association functional class III or IV congestive heart failure in the Beta-Blocker Evaluation Survival Trial (BEST) revealed that 10% had the V122I variant (11,18).

The relatively low median age of our cohort (52 in individuals with P/LP *TTR* variants)—particularly the African-ancestry subset—likely explains the low reported rates of observed disease in the full cohort analysis (49). That is, many of the individuals may still be in a “pre-symptomatic” stage of disease development based on age. Indeed, in the age-stratified subanalysis (≥60 years), we observed a marked increase in the odds of heart disease, including cardiomyopathy, heart failure, and atrial fibrillation, in individuals with P/LP *TTR* variants. These findings are thus very similar to the prior work of Buxbaum et al (21) in the ARIC and CHS cohorts, as well as the more recent analysis of Damrauer et al (9) in the Penn Medicine Biobank, both showing that V122I was associated with increased odds of heart failure with advanced age (65 years and 50 years, respectively). Our findings were not specific to V122I alone, as later-onset cardiomyopathy was also observed in association with V30M, T60A, and I68L.

### Alternative explanations for limited disease associations

Nonpenetrance is also a factor in explaining our findings. Incomplete (if not low) penetrance has been a consistent finding in numerous genome-first inherited cardiomyopathy studies, so a similar pattern for *TTR* is possible (26,27). Despite an increased OR, only 12% and 22% of carriers ≥60 years of age were observed to have cardiomyopathy and heart failure, respectively, in our analysis. These data are consistent with a longitudinal analysis of individuals with V122I from ARIC, which reported 29% incidence of heart failure and fewer than 7% with incidence of hypertrophy and an infiltrative phenotype by echocardiography after 21.5 years of follow-up (12).

Under-recognition of ATTR also likely contributed to the limited associations observed from this retrospective analysis. Unfamiliarity with the disease—particularly outside of specialized centers—is known to present challenges to proper diagnosis and treatment (28,29). Patients may present with any number of nonspecific and heterogeneous symptoms, including those that arise from peripheral neuropathy; autonomic neuropathy; heart failure; and dysfunctions of the eye, kidneys, thyroid, adrenal glands, and blood vessels (30,31).

### Absence of association with polyneuropathy

The contributions of amyloidosis polyneuropathy to the observed associations in our analysis were limited. Moreover, nonspecific neuropathy associations were absent. Several factors may contribute to these observations. First, the majority of individuals we observed with P/LP *TTR* variants (137 of 157 patients; 87%) had a variant associated with a predominantly cardiac phenotype (T60A, I68L, V122I) (20,32). For example, the representation of V122I carriers enrolled in the clinical trials for hATTR amyloid polyneuropathy was very limited—only 2 of 225 patients in the patisiran trial and 3 of 172 patients in the inotersen trial (33,34). Similarly, among 92 individuals with V122I variants and a clinical diagnosis of heart failure in the study by Damrauer *et al* (9), only 24 (26%) had neuropathy. The proportion was higher in those with confirmed hATTR amyloid cardiomyopathy (5 of 10; 50%) (9). Secondly, it is also possible that the presence of polyneuropathy may remain undiagnosed. As many as 80% of hATTR patients display some degree of cardiac involvement, the degree of which is generally considered to be the major determinant of outcome (35, 36, 37). Hence, clinical presentation of heart disease may mask the related identification of neurologic phenotypes. Third, ICD code–based ascertainment of neuropathic phenotypes may be insensitive due to the clinical variability in the manifestations of polyneuropathy and the imperfect nature of documentation in the medical record.

### Implications for population genomic screening

Hereditary ATTR has historically been underdiagnosed, remaining a significant burden to families for generations (38). A population-based genome-first approach for identification of P/LP *TTR* gene variants has the potential to diagnose cases of previously unrecognized ATTR and identify patients at risk for developing hATTR cardiomyopathy or polyneuropathy. Our results support an association between heart disease and P/LP *TTR* variants in patients ≥60 years of age, particularly in populations with high prevalence of V122I. However, this approach to *TTR*-variant screening poses several challenges for management of asymptomatic patients who have no known family history of hATTR as there is no firmly established management or treatment guideline for asymptomatic individuals with P/LP *TTR* variants. A recent consensus statement recommends annual follow-up 10 years before an established predicted age of onset of symptomatic disease, which depends not only on the typical phenotype manifestation and age of onset for the particular variant but also the age of onset in any family members with a history of hATTR amyloidosis (39). However, such guidelines were designed with a typical symptoms-based case ascertainment in mind, so the applicability to individuals identified via genomic screening must be defined. The cost of current U.S. Food and Drug Administration–approved therapies may be prohibitive and difficult to justify in asymptomatic individuals with subclinical or no evidence of subclinical disease (40). This is particularly true if a large number of individuals are identified through a large genomics-based approach, such as MyCode. Furthermore, we do not know at which point to initiate therapy in these patients, or if alternative treatment strategies with off-label agents would be appropriate.

Regardless of these challenges, there is a clear and timely need for systematic research to answer these remaining questions. Several existing therapies have come to market in recent years and several more promising candidates are in development, increasing potential opportunities to improve patient outcomes with appropriate ATTR diagnosis. Furthermore, the prevailing trends in genomics—broader use at earlier ages—are likely to remain, such that the number of individuals identified with P/LP *TTR* variants will continue to grow in the coming years, increasing the need for evidence-based guidelines.

### Study limitations

Our analysis was limited by relying on EHR data for defining phenotypes. As such, the true disease burden is likely higher than reported because of a limited ability to characterize the presence of disease in the absence of clinical symptoms. As discussed above, this limitation may be especially challenging for the detection of ATTR polyneuropathy.

Furthermore, our analysis is limited in the relative underrepresentation of individuals of African ancestry in our MyCode cohort (2.6%)—in whom the V122I variant is more common. However, the absolute size of this African-ancestry subset (n = 3,368) compares favorably to other cohorts (eg, Penn Medicine Biobank [n = 3,724], and ARIC [n = 3,856]) (9,12). The age characteristics of our African-ancestry subset were younger than the typical age of onset for V122I-associated cardiomyopathy, so longitudinal follow-up will be required to definitively determine penetrance.

## Conclusions

In a large population-based US health care cohort with exome sequencing data, we found that individuals with P/LP variants in *TTR* identified by genomic screening had significantly increased odds of heart disease (including heart failure, cardiomyopathy, and atrial fibrillation) on or after the age of 60 years. In at least 14% of these cases, this diagnosis of heart disease coincided with LV septal thickening—substantially raising suspicion of ATTR—even though diagnosis of amyloidosis was only present in 1 case, providing evidence of disease underdiagnosis.

Our results provide a proof-of-concept for a genome-first approach to the identification of hATTR cardiomyopathy and help to inform the potential inclusion of the *TTR* gene in recommendations on secondary findings reporting and population-based genomic screening analyses. With the advent of less-expensive and broader genomic sequencing on a population level, the number of individuals identified as at risk for hATTR will continue to increase. Prospective studies leveraging data with the ability to recontact patients to gather ongoing clinical phenotype data will be essential to developing appropriate surveillance and early treatment strategies in these individuals and their families, particularly given the clear impact of age on disease penetrance.

Perspectives **COMPETENCY IN MEDICAL KNOWLEDGE:** Genetic variants in transthyretin, particularly the V122I variant, are associated with an increased risk of heart disease later in life. Hereditary transthyretin amyloidosis is likely an underdiagnosed cause of that disease. Genome-first approaches may help to improve recognition and diagnosis of transthyretin amyloidosis.

**TRANSLATIONAL OUTLOOK:** Further research is needed in this area to determine the best approach to clinical surveillance and management of asymptomatic individuals, as well as the appropriate timing for initiation of treatment. Additionally, further research is needed to define the effectiveness of genome-first approaches in this setting.

## Funding Support and Author Disclosures

Supported by Geisinger and Regeneron Genetics Center. Dr Carry has stock ownership of Invitae Corp. All other authors have reported that they have no relationships relevant to the contents of this paper to disclose.

## References

[1] Ruberg F.L., Berk J.L. (2012). Transthyretin (TTR) cardiac amyloidosis. Circulation.

[2] Nativi-Nicolau J., Maurer M.S. (2018). Amyloidosis cardiomyopathy. Curr Opin Cardiol.

[3] Benson M., Scriver C., Beaudet A., Valle D. (2000). The Metabolic and Molecular Bases of Inherited Disease.

[4] Rintell D., Heath D., Braga Mendendez F. (2021). Patient and family experience with transthyretin amyloid cardiomyopathy (ATTR-CM) and polyneuropathy (ATTR-PN) amyloidosis: results of two focus groups. Orphanet J Rare Dis.

[5] Soper E.R., Suckiel S.A., Braganza G.T., Kontorovich A.R., Kenny E.E., Abul-Husn N.S. (2021). Genomic screening identifies individuals at high risk for hereditary transthyretin amyloidosis. J Pers Med.

[6] Maurer M.S., Schwartz J.H., Gundapaneni B. (2018). Tafamidis treatment for patients with transthyretin amyloid cardiomyopathy. N Engl J Med.

[7] Clinical Genome Resource https://actionability.clinicalgenome.org/ac/Adult/ui/stg2SummaryRpt?doc=AC145&version=24085.

[8] Miller D.T., Lee K., Chung W.K. (2021). ACMG SF v3.0 list for reporting of secondary findings in clinical exome and genome sequencing: a policy statement of the American College of Medical Genetics and Genomics (ACMG). Genet Med.

[9] Damrauer S.M., Chaudhary K., Cho J.H. (2019). Association of the V122I hereditary transthyretin amyloidosis genetic variant with heart failure among individuals of African or Hispanic/Latino ancestry. JAMA.

[10] Jacobson D.R., Pastore R.D., Yaghoubian R. (1997). Variant-sequence transthyretin (isoleucine 122) in late-onset cardiac amyloidosis in black Americans. N Engl J Med.

[11] Buxbaum J., Jacobson D.R., Tagoe C. (2006). Transthyretin V122I in African Americans with congestive heart failure. J Am Coll Cardiol.

[12] Quarta C.C., Buxbaum J.N., Shah A.M. (2015). The amyloidogenic V122I transthyretin variant in elderly black Americans. N Engl J Med.

[13] Landrum M.J., Lee J.M., Benson M. (2018). ClinVar: improving access to variant interpretations and supporting evidence. Nucleic Acids Res.

[14] Staples J., Maxwell E.K., Gosalia N. (2018). Profiling and leveraging relatedness in a precision medicine cohort of 92,455 exomes. Am J Hum Genet.

[15] Wang K., Li M., Hakonarson H. (2010). ANNOVAR: functional annotation of genetic variants from high-throughput sequencing data. Nucleic Acids Res.

[16] Firth D. (1993). Bias reduction of maximum likelihood estimates. Biometrika.

[17] Staples J., Qiao D., Cho M.H., Silverman E.K., Nickerson D.A., Below J.E. (2014). PRIMUS: rapid reconstruction of pedigrees from genome-wide estimates of identity by descent. Am J Hum Genet.

[18] Buxbaum J.N., Ruberg F.L. (2017). Transthyretin V122I (pV142I)∗ cardiac amyloidosis: an age-dependent autosomal dominant cardiomyopathy too common to be overlooked as a cause of significant heart disease in elderly African Americans. Genet Med.

[19] Sattianayagam P.T., Hahn A.F., Whelan C.J. (2012). Cardiac phenotype and clinical outcome of familial amyloid polyneuropathy associated with transthyretin alanine 60 variant. Eur Heart J.

[20] Rapezzi C., Quarta C.C., Obici L. (2013). Disease profile and differential diagnosis of hereditary transthyretin-related amyloidosis with exclusively cardiac phenotype: an Italian perspective. Eur Heart J.

[21] Buxbaum J., Alexander A., Koziol J., Tagoe C., Fox E., Kitzman D. (2010). Significance of the amyloidogenic transthyretin Val 122 Ile allele in African Americans in the Arteriosclerosis Risk in Communities (ARIC) and Cardiovascular Health (CHS) studies. Am Heart J.

[22] Dungu J.N., Papadopoulou S.A., Wykes K. (2016). Afro-Caribbean heart failure in the United Kingdom. Circ Heart Fail.

[23] Lahuerta Pueyo C., Aibar Arregui M.Á., Gracia Gutierrez A., Bueno Juana E., Menao Guillén S. (2019). Estimating the prevalence of allelic variants in the transthyretin gene by analysing large-scale sequencing data. Eur J Hum Genet.

[24] Benson M.D., Wallace M.R., Tejada E., Baumann H., Page B. (1987). Hereditary amyloidosis: description of a new American kindred with late onset cardiomyopathy. Arthritis Rheum.

[25] Reilly M.M., Staunton H., Harding A.E. (1995). Familial amyloid polyneuropathy (TTR ala 60) in north west Ireland: a clinical, genetic, and epidemiological study. J Neurol Neurosurg Psychiatr.

[26] Carruth E.D., Young W., Beer D. (2019). Prevalence and electronic health record-based phenotype of loss-of-function genetic variants in arrhythmogenic right ventricular cardiomyopathy-associated genes. Circ Genomic Precis Med.

[27] Haggerty C.M., Damrauer S.M., Levin M.G. (2019). Genomics-first evaluation of heart disease associated with titin-truncating variants. Circulation.

[28] Lane T., Fontana M., Martinez-Naharro A. (2019). Natural history, quality of life, and outcome in cardiac transthyretin amyloidosis. Circulation.

[29] Alexander K.M., Orav J., Singh A. (2018). Geographic disparities in reported US amyloidosis mortality from 1979 to 2015 potential underdetection of cardiac amyloidosis. JAMA Cardiol.

[30] Maurer M.S., Bokhari S., Damy T. (2019). Expert consensus recommendations for the suspicion and diagnosis of transthyretin cardiac amyloidosis. Circ Heart Fail.

[31] Witteles R.M., Bokhari S., Damy T. (2019). Screening for transthyretin amyloid cardiomyopathy in everyday practice. J Am Coll Cardiol HF.

[32] Maurer M.S., Hanna M., Grogan M. (2016). Genotype and phenotype of transthyretin cardiac amyloidosis: THAOS (Transthyretin Amyloid Outcome Survey). J Am Coll Cardiol.

[33] Adams D., Gonzalez-Duarte A., O’Riordan W.D. (2018). Patisiran, an RNAi therapeutic, for hereditary transthyretin amyloidosis. N Engl J Med.

[34] Benson M.D., Waddington-Cruz M., Berk J.L. (2018). Inotersen treatment for patients with Hereditary transthyretin amyloidosis. N Engl J Med.

[35] Maurer M.S., Elliott P., Comenzo R., Semigran M., Rapezzi C. (2017). Addressing common questions encountered in the diagnosis and management of cardiac amyloidosis. Circulation.

[36] Maurer M.S., Hanna M., Grogan M. (2016). Genotype and phenotype of transthyretin cardiac amyloidosis. J Am Coll Cardiol.

[37] Ruberg F.L., Grogan M., Hanna M., Kelly J.W., Maurer M.S. (2019). Transthyretin amyloid cardiomyopathy. J Am Coll Cardiol.

[38] Galant N.J., Westermark P., Higaki J.N., Chakrabartty A. (2017). Transthyretin amyloidosis: an under-recognized neuropathy and cardiomyopathy. Clin Sci.

[39] Conceição I., Damy T., Romero M. (2019). Early diagnosis of ATTR amyloidosis through targeted follow-up of identified carriers of TTR gene mutations∗. Amyloid.

[40] Kazi D.S., Bellows B.K., Baron S.J. (2020). Cost-effectiveness of tafamidis therapy for transthyretin amyloid cardiomyopathy. Circulation.

