# Supplementary material for: Genomic Screening for Pathogenic Transthyretin Variants Finds Evidence of Underdiagnosed Amyloid Cardiomyopathy From Health Records
Source: JACC CardioOncol. 2021 Oct 19;3(4):550–61. doi: 10.1016/j.jaccao.2021.07.002 (PMC8543083; doi:10.1016/j.jaccao.2021.07.002)
Supplement: Supplemental Data [file mmc1.pdf]

SUPPLEMENTAL MATERIAL

Supplemental Table 1. Diagnosis codes used for phenotype definitions

**Phenotype**	**ICD or Epic† Codes***
Cardiac Amyloid	152733, 152730, 225871
Amyloid, General	E85.0, E85.1, E85.2, E85.3, E85.4, E85.89, E85.9, 277.39, 277.30, 277.3
Aortic Valve Stenosis	I35.0, I35.2, 424.1
Atrial Fibrillation	Custom phenotype(1)
Atrioventricular Block	I44.0, I44.1, I44.2, I44.3, I44.39, 426.10, 426.11, 426.12, 426.13
Autonomic Dysfunction‡	I95.1, G90.01, G90.09, G90.4, G90.8, G90.9, G99.0, K34.81, L74.9, 337.00, 337.01, 337.09, 337.1, 337.3, 337.9, 458.0, 536.3, 705.9)
Bundle Branch Block	I44.7, I45.10, I45.19, 426.2, 426.3, 426.4, 426.50, 426.51, 426.52, 426.53, 426.54
(Non-ischemic) Cardiomyopathy	I42.1, I42.2, I42.5, I42.8, I42.9, I43, 425.11, 425.18, 425.4, 425.7, 425.8, 425.9
Heart Failure	Custom phenotype(2)
Sick Sinus Syndrome	I49.5, 427.81
Carpal Tunnel Syndrome	G56.00, G56.01, G56.02, G56.03, 354.0
Cataracts	H25.011, H25.012, H25.013, H25.019, H25.031, H25.032, H25.033, H25.039, H25.041, H25.042, H25.043, H25.049, H25.091, H25.092, H25.093, H25.099, H25.10, H25.11, H25.12, H25.13, H25.20, H25.21, H25.22, H25.23, H25.811, H25.812, H25.813, H25.819, H25.89, H25.9, H26.20, H26.211, H26.212, H26.213, H26.219, H26.221, H26.222, H26.223, H26.229, H26.231, H26.232, H26.233, H26.239, H26.40, H26.491, H26.492, H26.493, H26.499, H26.8, H26.9, H28, 366.10, 366.11, 366.12, 366.13, 366.14, 366.15, 366.16, 366.17, 366.18, 366.19, 366.30, 366.31, 366.32, 366.33, 366.34, 366.8, 366.9
Glaucoma	H40.11, H40.15, H40.5, 365.11, 365.15, 365.6
Hepatomegaly	R16.0, R16.2, 789.1
Impotence	F52.21, F52.22, F52.8, R37, N52.1, N52.8, N52.9, 302.72, 607.84
Incontinence	N39.3, N39.41, N39.42, N39.43, N39.44, N39.45, N39.46, N39.490, N39.491, N39.492, N39.498, N39.8, N39.9, R32, 599.89, 599.9, 625.6, 788.30, 788.31, 788.32, 788.33, 788.34, 788.35, 788.36, 788.37, 788.38, 788.39

Limb mononeuropathy or unspecified mononeuropathy/polyneuropathy	G56.9, G57.9, G58.9, G62.9, 354.9, 355.8, 355.9, 357.9
Spinal Stenosis	M48.0, 724.0

*Code required to either be present on patient problem list or ≥2 encounters to be valid †Epic grouper codes used for outpatient encounters used for specific diagnosis of ‘Cardiac Amyloid’; all other codes are ICD-9 or ICD-10. ‡Includes gastroparesis, orthostatic hypotension, and eccrine sweat disorder

Supplemental Table 2. Observed P/LP variants in *TTR* in individuals ≥60 years of age in MyCode

**Variant**	**HGVSc**	**HGVSp**	**Number of Individuals**
V30M	NM_000371:c.148G>A	p.Val50Met	6
L58H	NM_000371:c.233T>A	p.Leu78His	1
T60A	NM_000371:c.238A>G	p.Thr80Ala	4
I68L	NM_000371:c.262A>T	p.Ile88Leu	8
V122I	NM_000371:c.424G>A	p.Val142Ile	30

Regeneron Genetics Center banner author list and contribution statements

All authors/contributors are listed in alphabetical order.

<u>RGC Management and Leadership Team</u>

Goncalo Abecasis, Ph.D., Aris Baras, M.D., Michael Cantor, M.D., Giovanni Coppola, M.D., Aris Economides, Ph.D., Luca A. Lotta, M.D., Ph.D., John D. Overton, Ph.D., Jeffrey G. Reid, Ph.D., Alan Shuldiner, M.D.

Contribution: All authors contributed to securing funding, study design and oversight. All authors reviewed the final version of the manuscript.

<u>Sequencing and Lab Operations</u>

Christina Beechert, Caitlin Forsythe, M.S., Erin D. Fuller, Zhenhua Gu, M.S., Michael Lattari, Alexander Lopez, M.S., John D. Overton, Ph.D., Thomas D. Schleicher, M.S., Maria Sotiropoulos Padilla, M.S., Karina Toledo, Louis Widom, Sarah E. Wolf, M.S., Manasi Pradhan, M.S., Kia Manoochehri, Ricardo H. Ulloa.

Contribution: C.B., C.F., K.T., A.L., and J.D.O. performed and are responsible for sample genotyping. C.B, C.F., E.D.F., M.L., M.S.P., K.T., L.W., S.E.W., A.L., and J.D.O. performed and are responsible for exome sequencing. T.D.S., Z.G., A.L., and J.D.O. conceived and are responsible for laboratory automation. M.P., K.M., R.U., and J.D.O are responsible for sample tracking and the library information management system.

<u>Genome Informatics</u>

Xiaodong Bai, Ph.D., Suganthi Balasubramanian, Ph.D., Leland Barnard, Ph.D., Andrew Blumenfeld, Gisu Eom, Lukas Habegger, Ph.D., Alicia Hawes, B.S., Shareef Khalid, Jeffrey G. Reid, Ph.D., Evan K. Maxwell, Ph.D., William Salerno, Ph.D., Jeffrey C. Staples, Ph.D., Ashish Yadav, M.S.

Contribution: X.B., A.H., W.S. and J.G.R. performed and are responsible for analysis needed to produce exome and genotype data. G.E. and J.G.R. provided compute infrastructure development and operational support. S.K., S.B., and J.G.R. provide variant and gene annotations and their functional interpretation of variants. E.M., L.B., J.S., A.B., A.Y., L.H., J.G.R. conceived and are responsible for creating, developing, and deploying analysis platforms and computational methods for analyzing genomic data.

<u>Research Program Management</u>

Marcus B. Jones, Ph.D., Lyndon J. Mitnaul, Ph.D.

Supplemental References

1. Raghunath S, Pfeifer JM, Cerna AEU, et al. Deep Neural Networks can Predict New-Onset Atrial Fibrillation from the 12-lead Electrocardiogram and Help Identify Those at Risk of AF-Related Stroke. Circulation 2021;(In Press).

2. Jing L, Ulloa Cerna AE, Good CW, et al. A Machine Learning Approach to Management of Heart Failure Populations. JACC Hear. Fail. 2020;8:578–587.
